# Prenatal diagnosis of fetal skeletal dysplasia using 3-dimensional computed tomography: a prospective study

**DOI:** 10.1186/s12891-020-03663-x

**Published:** 2020-10-08

**Authors:** Miyoko Waratani, Fumitake Ito, Yukiko Tanaka, Aki Mabuchi, Taisuke Mori, Jo Kitawaki

**Affiliations:** Department of Obstetrics and Gynecology, Kyoto Prefectural University of Medicine, 645 Kaijii-cho, Kamigyo-ku, Kyoto, 602-8566 Japan

**Keywords:** Fetal skeletal dysplasia, Fetal diagnosis, 3D-CT, Shortening of long bones, Gene expression

## Abstract

**Background:**

Fetal skeletal dysplasia (FSD) comprises a complex group of systemic bone and cartilage disorders. Many FSD phenotypes have indistinct definitions, making definitive prenatal diagnosis difficult. The condition is typically diagnosed using sonography; however, three-dimensional computed tomography (3D-CT) also aids in making a prenatal diagnosis. This study aimed to examine the efficacy of 3D-CT in the prenatal diagnosis of FSD by comparing the diagnostic accuracy of fetal sonography and 3D-CT.

**Methods:**

On suspicion of FSD based on ultrasound examination, we performed 3D-CT prenatally to obtain detailed skeletal information on FSD. To minimize exposure of the fetuses to radiation without compromising image quality, we used predetermined 3D-CT settings for volume acquisition.

**Results:**

Nineteen fetuses were suspected of having skeletal dysplasia based on ultrasonography findings. Of these, 17 were diagnosed with FSD using 3D-CT. All 17 fetuses diagnosed with FSD prenatally were confirmed postnatally to have the condition. The postnatal diagnosis (campomelic dysplasia) differed from the prenatal diagnosis (osteogenesis imperfecta) in only one infant. Sixteen cases (94.1%) were diagnosed both prenatally and postnatally with FSD. Five infants had lethal skeletal dysplasia; one died in utero, and four died as neonates. We determined the appropriate delivery method for each infant based on the prenatal diagnosis.

**Conclusions:**

3D-CT is a valuable tool for augmenting ultrasound examinations in the diagnosis of FSD. While improving the diagnostic tool of sonography is essential in cases of suspected FSD, 3D-CT imaging is indispensable for diagnosis and classification, enabling better planning for resuscitation of the infant after birth.

**Trial registration:**

University Hospital Medical Information Network (UMIN) Center trial registration number is UMIN000034744. Registered 1 October, 2018 – Retrospectively registered.

## Background

Fetal skeletal dysplasia (FSD) is a group of systemic bone and cartilage disorders that develops prenatally and may be detected by fetal ultrasonography. Considering most cases of skeletal dysplasia involve the mutation of a single gene, a postnatal diagnosis can be reached if this mutation is identified. Although the widespread use of fetal ultrasound imaging has improved the detection of FSD, prenatal diagnosis remains relatively difficult because of the rarity of the condition (approximately one in every 5000 births) [1]. The diversity of the FSD phenotypes also contributes to the difficulty in diagnosing these disorders. FSDs comprise 436 diseases that are classified into 42 groups, according to the Nosology & Classification of Genetic Skeletal Disorders: 2015 Revision [2]; however, the phenotypic definition of each disease is often unclear. It is important to clarify whether it is a lethal or nonlethal disorder to plan the most effective delivery method and the best postnatal treatment [3].

Fetal ultrasonography is the most standard method to detect FSD; however, it is unsuitable for the analysis of the skeleton because supersonic waves reflect it with the bone cortex. It was reported that the rate of accurate prenatal diagnosis of skeletal dysplasia was approximately 65% [4]. Fetal magnetic resonance imaging (MRI) is useful for the analysis of the fetal spine and depiction of marrow and soft tissue; however, it is unsuitable for bone cortical depiction like fetal sonography. Three-dimensional computed tomography (3D-CT) is commonly used to diagnose cardiac atherosclerosis, cerebral aneurysms, and cerebral artery stenosis. This imaging technique creates better images of thromboses and vascular wall calcification in cerebral aneurysms compared to cerebral angiography. In 2004, Ruano et al. reported that fetal 3D-CT was a useful diagnostic method, complementary to ultrasonography, and may improve the diagnostic accuracy of skeletal disorders [5]. Considering the increased risk associated with the radiation introduced by this clinically beneficial method, we elected to use low-dose 3D-CT for prenatal diagnosis in this study. This study aimed to determine the efficacy of 3D-CT in the prenatal diagnosis of FSD by comparing the prenatal and postnatal diagnoses in patients with this condition.

## Methods

### Study design

A prospective study was conducted involving pregnant women whose pregnancy/childbirth was being managed by the Department of Obstetrics and Gynecology at the Kyoto Prefectural University of Medicine during a period from 2012 to 2019. Fetuses were included in the study if they had a suspected bone abnormality based on routine ultrasound examinations. We recorded any family history of skeletal dysplasia and complications during pregnancy.

### Materials

Pregnant women whose pregnancies/childbirths were managed at our hospital and who met the following selection criteria were included as participants: (1) pregnancy at age 20 years or older at the time of consent, (2) gestational age > 24 weeks of pregnancy, (3) fetal ultrasonography showing a short femur (below the 10th percentile of fetal measurements) or an image of a fracture, curvature, or deformity in a long bone, skull, rib, or spine, (4) written informed consent to undergo 3D-CT was provided.

### Sonographic examination

We used Voluson E8 and E10 ultrasound systems (General Electric Healthcare, Tokyo, Japan) to screen for associated anomalies. All operators were experts in fetal sonography who have the certification of a perinatal specialist with an experience of more than seven years. We began by measuring the fetus using a standard approach (biparietal diameter, abdominal circumference, and femur length) and identifying fetuses that displayed femur length measurements below the 10th percentile. We then recommended 3D-CT scanning if the fetal femur or any other long bones were found to be shortened, regardless of whether they were abnormally shaped, as this indicated potential FSD.

### Three-dimensional computed tomography

3D-CT (Aquilion RXL; TOSHIBA, Tokyo, Japan) was only performed after thoroughly explaining the potential effects of radiation exposure to the mother and receiving informed consent from the mother. To minimize exposure of the fetus to radiation without compromising image quality, the following predetermined settings (Table 1) were used for volume acquisition.

**Table 1:** Protocol of 3D-CT

slice thickness (mm)	1.0
slice interval (mm)	1.0
rotation time (sec)	0.5
helical pitch	1.438
voltage (kV)	120
beam width (mm)	16
CTDI_vol_ (mGy) mean (range)	3.5 (1.9–4.9)
DLP (mGy.cm) mean (range)	133.6 (58.1–175.8)

*CTDI*_*vol*_ CT dose index volume, *DLP* dose length product

This was low-dose 3D-CT, a median CT dose index volume (CTDI_vol_) of 3.5 mGy (range: 1.9–4.9 mGy), that we reduced to approximately 30% of radiation doses to be required in the case of an examination for standard dose of abdominal CT. We created a maximum intensity projection (MIP) image from 3D-CT images for diagnosis, if necessary. The MIP is a method to make an X-ray image where we are able to take an image focused only on the area of main observation from 3D-CT imaging. Additional tests, such as MRI or amniotic chromosomal testing, were performed if required to determine fetal prognosis or to differentiate skeletal dysplasia from other diseases. Lethality was determined by the presence of a narrow thorax and lung hypoplasia based on ultrasonograms and MRI images.

### Postnatal examination

Postnatally, we recorded the neonatal weight and length and performed an X-ray examination to verify the shape of all bones. In the cases where genetic examination was needed, testing was performed to confirm the diagnosis after obtaining informed consent from the parents. Genomic DNA was extracted from the umbilical cord or the infant’s blood.

## Results

A total of 2447 fetuses underwent prenatal sonography and were delivered in our institution between 2012 and 2019. Of the 2447 fetuses, sonography revealed that 19 were suspected of having FSD, and we performed 3D-CT for those 19 fetuses. Of the 19 fetuses that underwent 3D-CT, 17 were diagnosed with FSD prenatally; however, the other two cases were diagnosed as not being FSD (one of the normal fetus, and another case of suspected trisomy 21). FSD was confirmed postnatally in all 17 of these fetuses. Sonography had a sensitivity of 100%, a specificity 99%, and positive and negative predictive values of 89.5 and 100%, respectively. On the other hand, 3D-CT had 100% sensitivity and specificity, and additionally, positive and negative predictive values of 100%.

### Patient characteristics

The mean maternal age was 32.2 (range: 20–41) years, and the mean gestational age at the first findings of FSD was 24.8 (range: 15–36) weeks. The mean 3D-CT imaging period was 31.1 (range: 24–38) weeks’ gestation, which is consistent with the period when CT testing is generally performed for prenatal diagnosis (approximately 30 weeks’ gestation). Patient characteristics are presented in Table 2.

**Table 2:** Characteristics of 17 infants with FSD

Infant	Maternal age (years)	GA at the first finding of FSD (weeks)	GA at 3D-CT (weeks)	GA at delivery (weeks)	Delivery mode	Birth weight (g)	Prognosis
1	33	36	36	38	CS	2599	alive
2	20	27	27	34	CS	2243	alive
3	30	30	30	36	BE	1587	1 day
4	38	15	28	37	CS (breech)	2169	3 days
5	27	24	24	37	VD	2233	2 days
6	30	24	28	37	CS	2924	alive
7	34	29	33	38	VD	3400	alive
8	40	25	33	38	VD	2621	alive
9	29	22	31	38	CS (repeat)	2250	alive
10	41	27	31	38	CS (NRFS)	2080	alive
11	29	33	35	38	VD	2496	alive
12	30	34	38	39	VD	2676	alive
13	39	17	35	37	CS	2569	alive
14	39	30	27	39	CS (repeat)	1843	alive
15	38	18	36	37	CS (breech)	2866	alive
16	25	16	27	27	VD	–	IUFD
17	26	15	28	38	CS (breech)	1710	1 day

*BE* breech extraction, CS: cesarean section, *GA* gestational age, *IUFD* intrauterine fetal death, *NRFS* non-reassuring fetal status, *FSD* fetal skeletal dysplasia, *VD* vaginal delivery

### Classification of fetal skeletal dysplasia

Prenatal imaging data, postnatal findings, and pre- and postnatal diagnosis are presented in Table 3. The specific postnatal diagnoses were as follows: osteogenesis imperfecta (OI, *n* = 3), thanatophoric dysplasia (TD, *n* = 2), achondroplasia/hypochondroplasia (ACH/HCH, *n* = 6), hypophosphatasia (HPP, *n* = 2), campomelic dysplasia (CD, *n* = 2), Desbuquois dysplasia (*n* = 1), and Creveld syndrome (*n* = 1). The two infants with HPP were siblings.

**Table 3:** Comparison between pre- and postnatal findings and diagnosis

Infant	Prenatal		Postnatal	
	findings	diagnosis	findings	diagnosis
1	Shortening and bowing of long bones, Fractures of femur and ribs	OI	Shortening and bowing of long bones, Fractures of femur and ribs	OI
2	Shortening and bowing of long bones, hydramnios	OI	Shortening and fractures of long bones	OI
3	Shortening and bowing of long bones, Narrow thorax, Megalencephaly	OI	Shortening and bowing of long bones, Rib fractures, Narrow thorax, Facial dysmorphy	OI
4	Shortening of long bones, Narrow thorax, Megalencephaly	TD	Rhyzomelic shortening of long bones, Bowing of long bones, Narrow thorax, Megalencephaly, Platyspondylia	TD
5	Shortening and bowing of long bones Long bones fractures Narrow thorax, Megalencephaly Hydramnios	TD	Shortening and bowing of long bones, Megalencephaly, Frontal bossing, Narrow thorax, Platyspondylia, Trident hand	TD
6	Shortening of long bones, Narrow thorax	ACH or HCH	Shortening of long bones, Narrow thorax	ACH
7	Shortening of long bones, Narrow thorax	ACH	Shortening of long bones, Narrow thorax	ACH
8	Shortening of long bones, Narrow thorax Trident hand	ACH	Shortening of long bones, Frontal bossing, Narrow thorax, Trident hand	ACH
9	Shortening of long bones, Narrow thorax	ACH	Shortening of long bones, Narrow thorax	ACH
10	Shortening of long bones, Narrow thorax, Trident hand	ACH	Shortening of long bones, Frontal bossing, Narrow thorax, Trident hand, Facial dysmorphy of Down syndrome	ACH Trisomy 21
11	Shortening of long bones	ACH or HCH	Shortening of long bones, Frontal bossing	HCH
12	Shortening and bowing of long bones Hypoplastic scapulae	CD	Shortening and bowing of long bones, Hypoplastic scapulae	CD
13	Shortening and bowing of long bones Mild curvature with scapular hypoplasia Club feet	**OI**	Bowing of long bones, Club feet, Hypoplastic scapulae, Rib fractures, Ambiguous genitalia, Facial dysmorphy	**CD**
14	Dislocation of elbow and knee, Club feet Radioulnar synostosis	Desbuquois disorder	Dislocation of shoulder, elbow, Articulation coxae and knee, Facial dysmorphy, Scoliosis, brevicollis	Desbuquois disorder
15	Shortening of long bones Hyperdactylia CHD (AVSD, single atrium)	Elis-van Creveld syndrome	Shortening of long bones, Narrow thorax, Hyperdactylia, CHD (AVSD, single atrium)	Elis-van Creveld syndrome
16	Shortening of long bones, Narrow thorax	HPP	–	HPP
17	Shortening of long bones, Narrow thorax, hydramnios	HPP	Shortening of long bones, Narrow thorax, Hypoplasia of lung, Low levels of serum ALP	HPP

*ACH* achondroplasia, *AVSD* atrial ventricular septal defect, *CD* campomelic dysplasia, *CHD* congenital heart disease, *HCH* hypochondroplasia, *HPP* hypophosphatasia, *OI* osteogenesis imperfecta, *TD* thanatophoric dysplasia

**Fig. 1:**
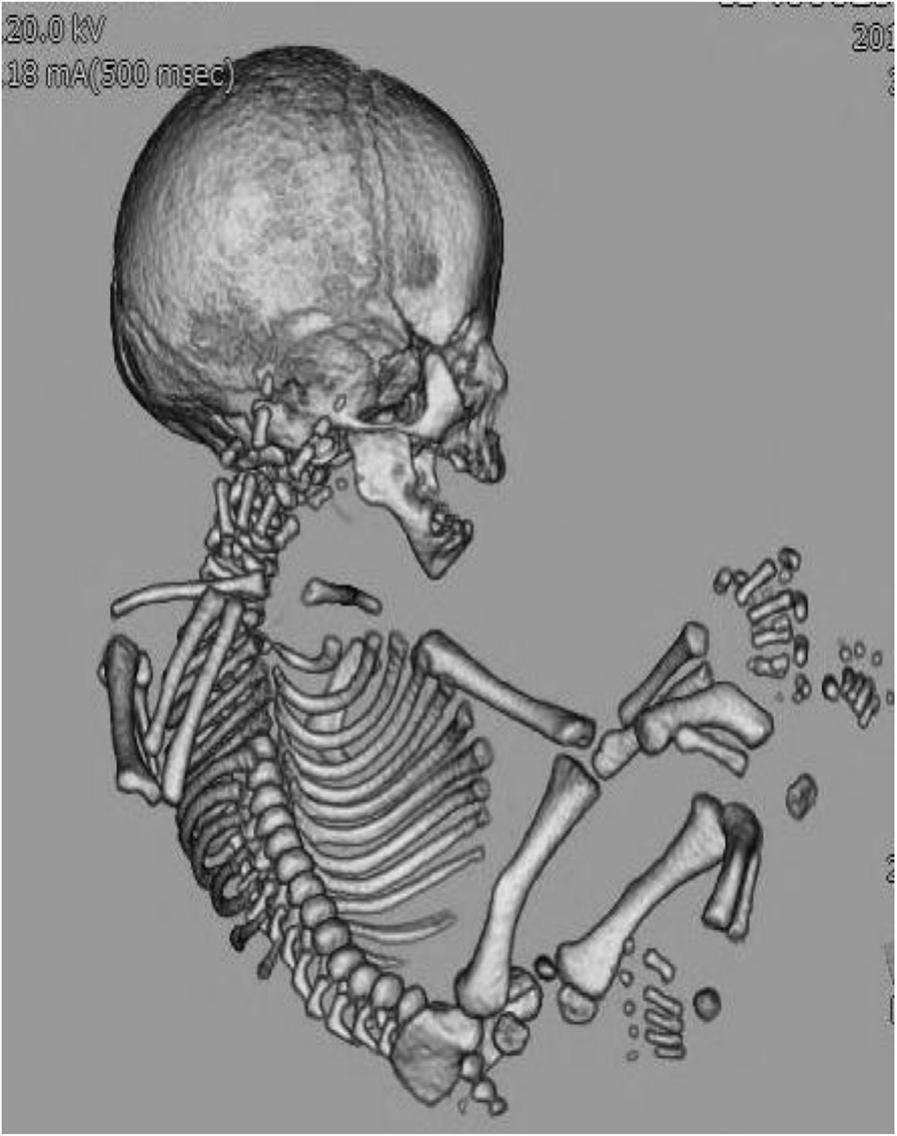
Fetal 3-dimensional computed tomography imaging of Infant 13: A case of campomelic dysplasia with a postnatal diagnosis that was different from the prenatal diagnosis

Sixteen infants (94.1%) were diagnosed both prenatally and postnatally with FSD. The pre- and post-natal diagnoses differed in one infant who was diagnosed with OI prenatally but with CD postnatally. In this case, prenatal findings by 3D-CT were long bone shortening and mild curvature with scapular hypoplasia (Fig. 1), therefore, we diagnosed “OI” prenatally. However, we diagnosed “CD” in the postnatal period because we identified ambiguous genitalia and facial dysmorphia.

### Lethality

Of the five fetuses with poor prognoses, one had OI, two had TD, and two had HPP. One of the fetuses with HPP suffered intrauterine fetal death at 27 weeks’ gestation, while the remaining four infants died within two days of birth. We identified lethality in the prenatal period in all five fetuses.

### Trisomy 21 and achondroplasia

**Fig. 2:**
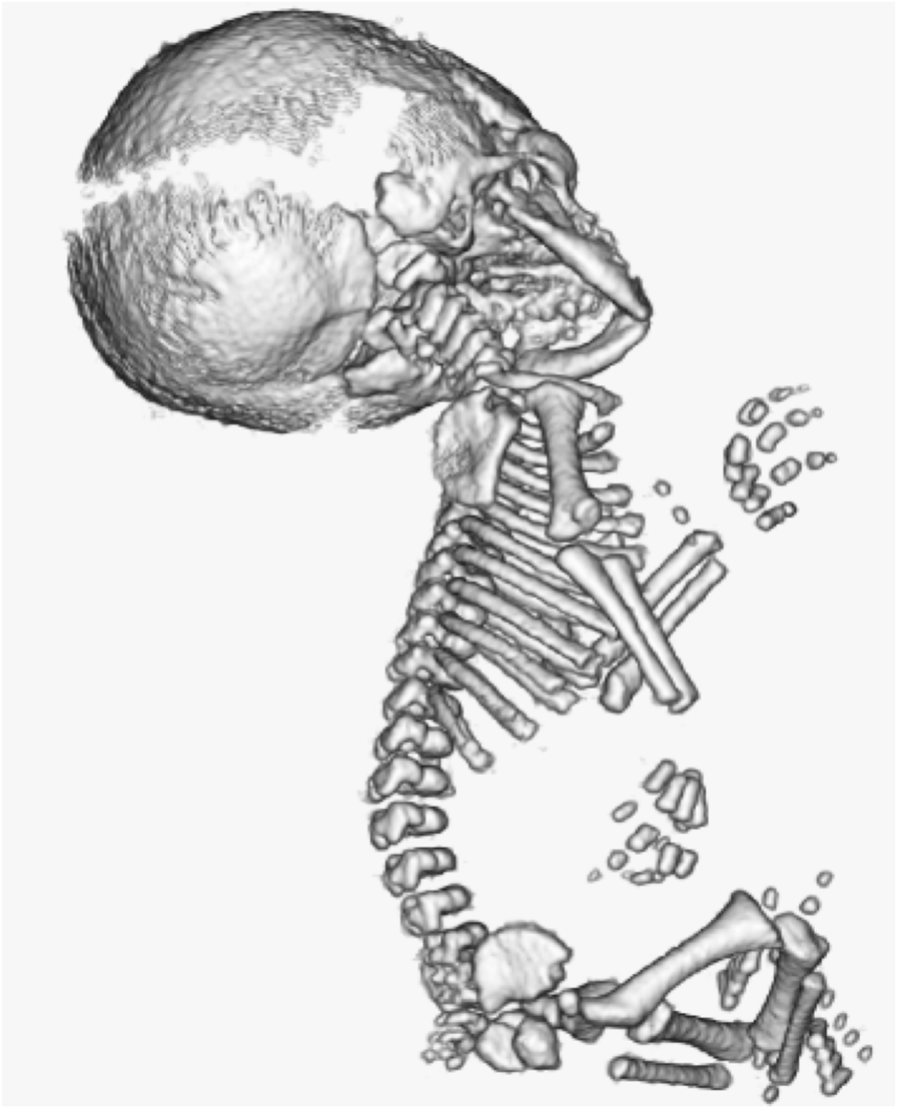
Fetal 3-dimensional computed tomography imaging of Infant 8: A case of achondroplasia with trisomy 21

Infant 8 was diagnosed prenatally with ACH based on ultrasound findings of shortened long bones, a narrow thorax, and a trident hand (Fig. 2). After birth, the characteristic trisomy 21 facial features were noted, and the results of chromosomal testing (G-band; 47, XY, + 21) confirmed trisomy 21. FGFR3 genetic analysis also indicated a G1193A mutation in G380, indicating that the infant also had ACH.

### Postnatal genetic testing

Genetic testing was performed in 11 infants (number; 1–2, 6–8, 10, 13–17).

**Fig. 3:**
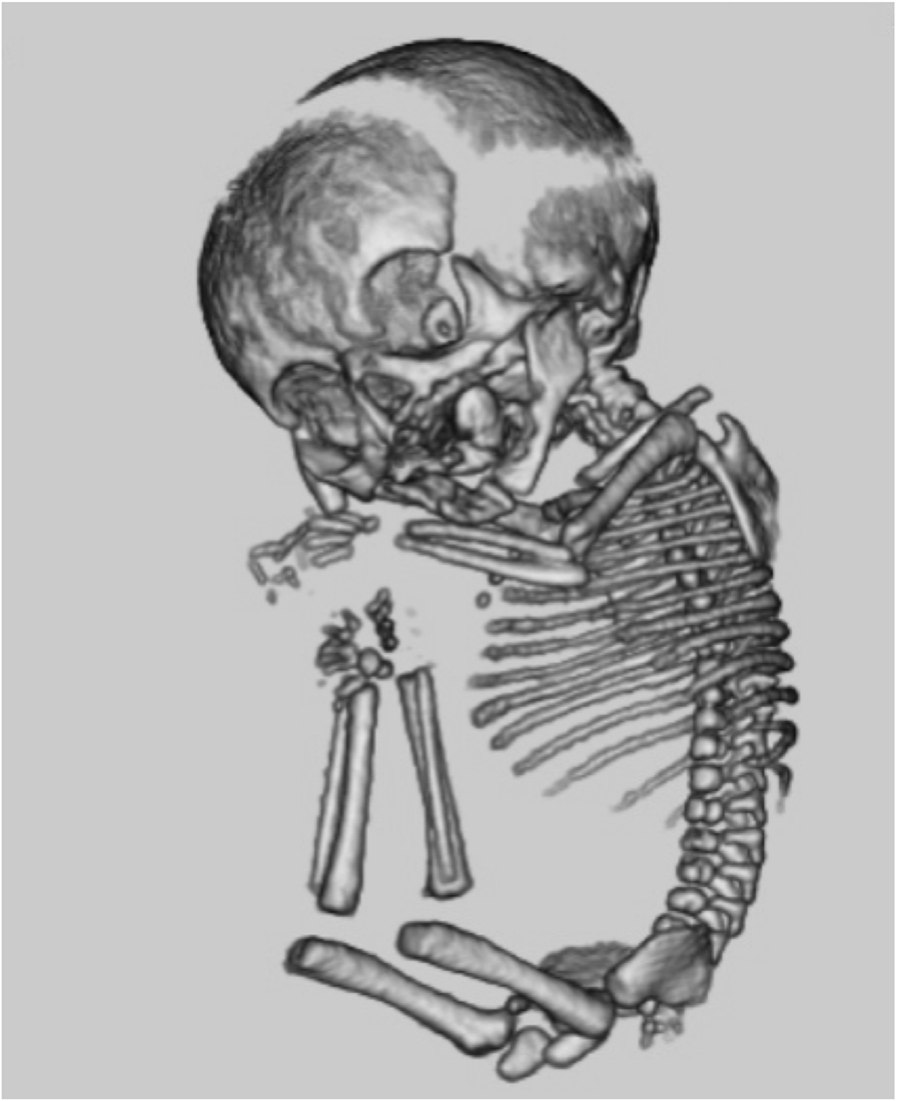
Fetal 3-dimensional computed tomography imaging of Infant 14: A case of Desbuquois dysplasia

In infant 12, we identified dislocation of the knee and elbow, as well as clubfeet, through sonography and 3D-CT, leading us to suspect Desbuquois dysplasia (Fig. 3). Postnatal genetic testing confirmed our suspicion of Desbuquois dysplasia (type 1) due to a mutation of the 613,165 calcium-activated nucleotidase 1 (CANT1) gene.

**Fig. 4:**
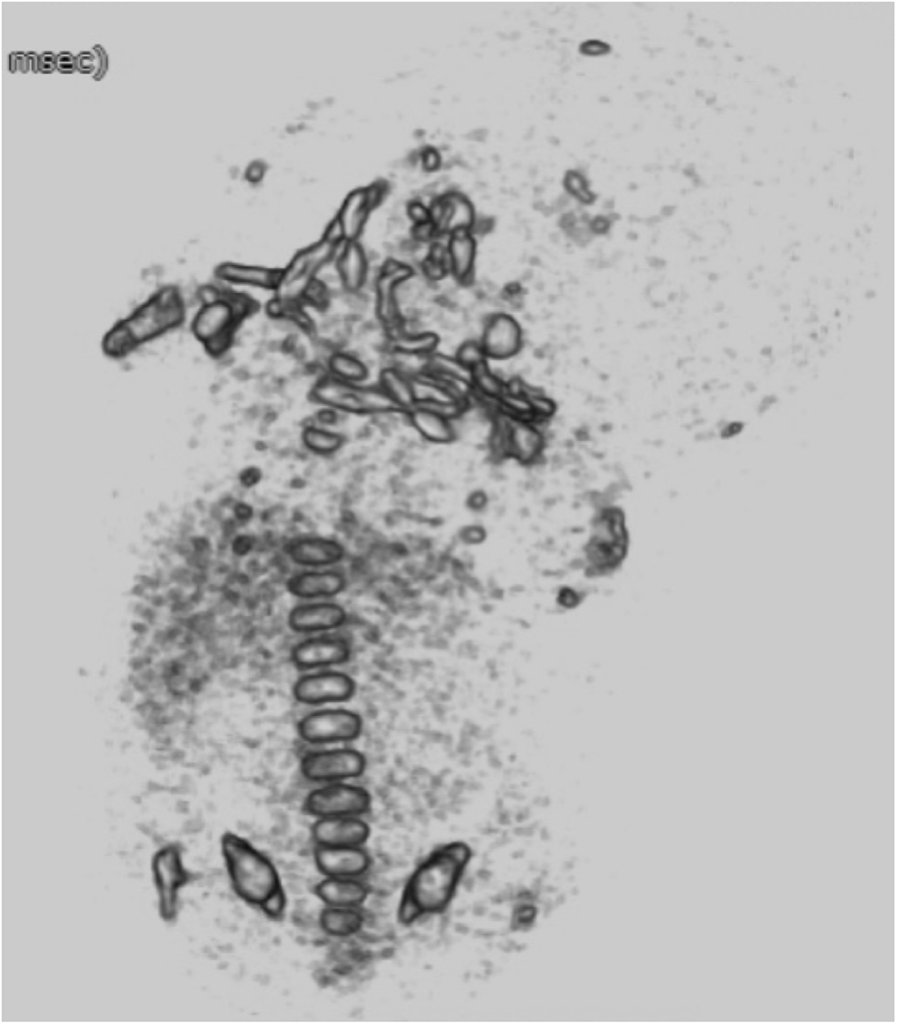
Fetal 3-dimensional computed tomography imaging of Infant 17: A case of hypophosphatasia

The two infants with HPP (infants 14 and 15) had the same parents. Ultrasonography at 18 weeks’ gestation in the first child revealed marked long bone shortening and a soft, deformed skull, which was suggestive of fetal HPP. Therefore, we measured maternal alkaline phosphatase (ALP) levels, which were low. At 27 weeks’ gestation, the child suffered an intrauterine fetal death. After the child was stillborn, autopsy imaging was performed. The CT images also indicated a likely diagnosis of HPP. Genetic testing of the placental tissue revealed a compound heterozygote with mutations in two tissue-nonspecific alkaline phosphatase genes (*ALPL*; p.K224E, c.1559delT), and confirmed our diagnosis of HPP. The parents’ second child exhibited severe shortening of all four limbs at 15 weeks’ gestation, with 3D-CT revealing findings typical of severe prenatal HPP (Fig. 4), with hardly any ossification. At 38 weeks’ gestation, the infant was delivered via cesarean section but suffered neonatal death at 1 day of age.

## Discussion

The first-line method for the prenatal diagnosis of skeletal dysplasia is typically sonographic imaging; however, it is difficult to perform a classification of FSD. Although 3D-CT may be a valuable imaging tool complementary to ultrasonography for the diagnosis and classification of FSD, it is necessary to select the appropriate sonography settings to minimize the degree of fetal irradiation without compromising image quality [6, 7]. Fetal radiation exposure should be concerned with fetal CT. The dose of 3D-CT was a mean CTDI_vol_ of 3.5 mGy (range: 1.9–4.9 mGy); this is “low-dose” fetal CT, which has been reported in 2013: a mean CTDI_vol_ of 2.2 mGy [4] and 4.2 mGy [8]. In 2008, the International Commission on Radiological Protection recommended that in utero exposure to less than 100 mGy would be of no practical significance and poses a lifetime cancer risk [9]. A mean CTDI_vol_ of 2.9 mGy is far below 100 mGy.

MRI may be useful to differentiate between FSD and other limb malformations [10, 11]. Spatial recognition is easy in fetal MRI, as the bone marrow and soft tissue are clearly identified. However, it may not be suitable for identifying the bone cortex. CT uses ionizing radiation to produce an X-ray image which is similar to that produced by plain radiography. Radiography has traditionally been used as a diagnostic imaging method for skeletal system diseases, and the experience can be reflected in diagnostic CT imaging using X-ray characteristics of skeletal system diseases. Observations from a 360° view are made possible by obtaining 3D images, and the diagnostic accuracy may be increased by observing from the direction with greatest visibility. Furthermore, fetuses in utero often have their limbs and spines flexed. Therefore, image reconstruction may be enabled by eliminating these limbs from view or selecting only the section to be observed by trimming, which then provides meaningful images for diagnosis [12]. Using low-dose 3D-CT imaging, we can create an MIP image of a point that we should focus on. The depiction of the details for abnormal points is made clearer by adding the MIP images; therefore, it helps in making a diagnosis of FSD.

Fetal bone 3D-CT (as an auxiliary test for ultrasound examinations) provides detailed information on systemic skeletal structure when performed at 26 weeks’ gestation onward. The detection of abnormal findings outside the skeletal system on sonography can greatly affect the diagnosis [8, 13]. For a diagnosis of FSD, both sonography and 3D-CT appear to be useful methods. In some cases, it can be difficult to differentiate skeletal dysplasia from a chromosomal abnormality. Of the 19 infants that underwent 3D-CT in our study, only one was suspected of having a chromosomal abnormality. In this case, skeletal dysplasia was suspected based on ultrasonography findings, but the results of 3D-CT suggested trisomy 21. Postnatal chromosomal testing was performed, resulting in a final diagnosis of trisomy 21 and the exclusion of this infant from the study. Some cases can be complicated, with concomitant skeletal dysplasia and a chromosome aberration. In particular, cases with combined ACH and trisomy 21 have been reported [14, 15]. ACH is included as a type of dysplasia in the group of FGFR3 abnormalities, where growing parts become hypoplastic due to endochondral ossification. This can be difficult to differentiate from trisomy 21 [14, 15]. In 2001 [16] and 2014 [17], it was reported that femoral shortening during the second trimester is associated with a high relative risk of chromosomal abnormalities, particularly trisomy 21. If fetal femoral shortening is observed, it is important to consider the possibility of conditions such as chromosomal abnormalities, fetal growth restriction, and various other anomalies. Attention should also be paid to the presence of abnormal findings outside of the skeletal system (e.g., abnormalities in cardiac structure, the nervous/gastrointestinal system, and facial features) to reach a differential diagnosis. When making a prenatal diagnosis, it is important to bear in mind that cases of concomitant skeletal dysplasia and chromosomal abnormalities are possible, albeit rare.

Desbuquois dysplasia is a severe autosomal recessive disorder that belongs to the multiple dislocation group [18]. It is characterized by shortened long bones, joint laxity, pre- and postnatal growth retardation, and progressive scoliosis. In the case of infant 12, the mother’s first child also had skeletal dysplasia (congenital dislocation of the knees), which was identified after birth. Although this child had been diagnosed with Larsen syndrome, it had neither been identified prenatally by sonography/3D-CT nor confirmed via postnatal genetic testing. The mother’s second child (infant 12), in contrast, underwent a prenatal ultrasound examination and 3D-CT and was diagnosed with Desbuquois dysplasia, rather than Larsen syndrome. Postnatal genetic testing led to a definitive diagnosis of Desbuquois dysplasia (type 1), which is due to a mutation in the *CANT1* gene [19, 20]. It is difficult to distinguish Desbuquois dysplasia and Larsen syndrome based on clinical manifestations. In the case of infant 12, it was demonstrated that an accurate prenatal diagnosis using an ultrasound examination and 3D-CT imaging was important to achieve a definitive diagnosis via postnatal molecular evaluation.

HPP is a condition in which tissue non-specific ALP loss or decrease causes abnormal bone and tooth mineralization [21]. Many cases of HPP are inherited in an autosomal recessive, rather than an autosomal dominant manner. HPP can be classified into the following six clinical forms, which vary based on severity [22]: 1) perinatal severe, 2) perinatal benign, 3) infantile, 4) childhood, 5) adult, and 6) odontohypophosphatasia. If parents have a child with HPP, they should be given genetic counseling with respect to future pregnancies, and the couple can decide whether to undergo carrier detection testing. If both the mother and father are carriers, the child has a 25% probability of being homozygous. Our fatal perinatal case appeared to be a severe one. The symptoms of perinatal cases appear early, resulting in intrauterine fetal death [23], stillbirth, or neonatal death. If we have a diagnosis of HPP in the prenatal period, we are able to start the enzyme replacement therapy promptly.

**Fig. 5:**
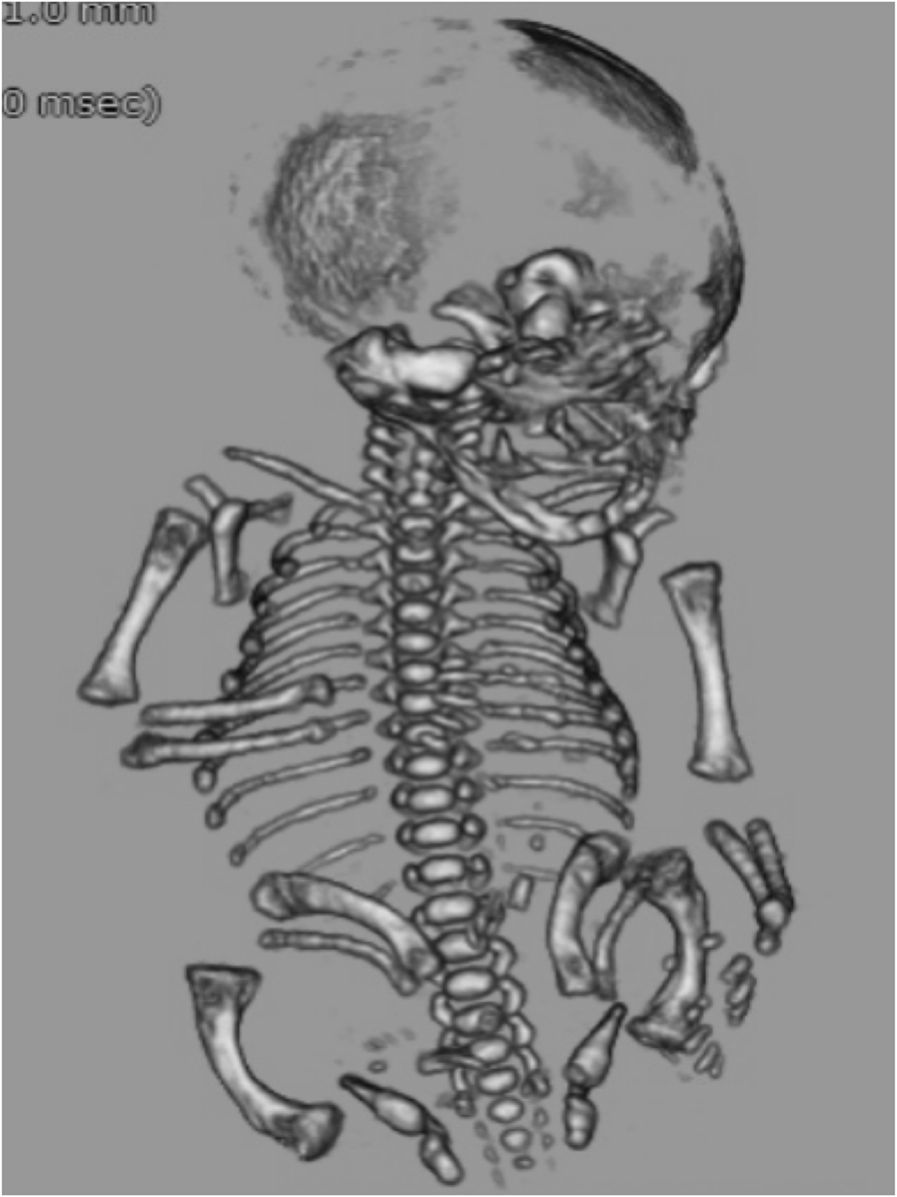
Fetal 3-dimensional computed tomography imaging of Infant 2: A case of osteogenesis imperfecta

Compared to normal fetuses, those with FSD are more likely to be in an abnormal fetal position owing to differences in physique, restricted posture, limited joint range of motion, and fractures [24]. In cases of OI, it can be difficult to prevent intrauterine fractures as they can be caused by fetal movement (Fig. 5). Studies have shown that, in cases of OI, performing a cesarean delivery does not increase the frequency of fetal bone fractures compared to vaginal delivery [25]. Moreover, in cases of the lethal type of OI, fetal survival does not appear to be affected by delivery mode (cesarean vs. vaginal delivery). For other types of skeletal dysplasias, the mode of delivery is often selected based on whether the condition is lethal. When the disease is diagnosed as lethal, vaginal delivery may be selected even if the fetus is breech. However, in cases with marked head enlargement, a cesarean section is required regardless of whether the case is fatal, owing to the potential difficulty in passing through the mother’s pelvis. Before delivery, the parents, obstetricians, pediatricians, and orthopedic surgeons need to discuss the plan for the resuscitation of the infant after birth. The classification by imaging of 3D-CT is imperative to decide on the delivery mode and treatment policy for the infant, for example, whether to perform endotracheal intubation, or to surgically treat the infants. Reliable prenatal diagnosis is required to determine whether the case is fetal and its degree of severity.

When considering the risk of radiation exposure, CT examination has not historically been the preferred method for pregnant women. However, in recent years, high-quality images adequate for diagnosis have been displayed by low-dose 3D-CT, reducing the level of radiation to which the mother and fetus are exposed. The limitation of this study is the relatively small sample size. Future studies should include a larger sample size to improve the power of the results. Continuation of this study is important to improve the diagnostic accuracy of fetal skeletal dysplasia.

## Conclusions

3D-CT is a valuable tool for augmenting ultrasound examinations in the diagnosis of FSD. While improving the diagnostic tool of sonography is important in cases of suspected FSD, 3D-CT imaging is indispensable for a diagnosis and a classification, enabling better planning for resuscitation of the infant after birth.

## Data Availability

The datasets generated and/or analyzed during this study are available in the University Hospital Medical Information Network (UMIN). (URL: https://upload.umin.ac.jp/cgi-open-bin/ctr_e/ctr_view.cgi?recptno=R000039610).

## Abbreviations

FSD: Fetal skeletal dysplasia
3D-CT: Three-dimensional computed tomography
MRI: Magnetic resonance imaging
OI: Osteogenesis imperfecta
TD: Thanatophoric dysplasia
ACH/HCH: Achondroplasia/hypochondroplasia
HPP: Hypophosphatasia
CD: Campomelic dysplasia
CANT1: Calcium-activated nucleotidase 1
ALP: Alkaline phosphatase
